# A Host KH RNA-Binding Protein Is a Susceptibility Factor Targeted by an RXLR Effector to Promote Late Blight Disease^☆^

**DOI:** 10.1016/j.molp.2015.04.012

**Published:** 2015-09-07

**Authors:** Xiaodan Wang, Petra Boevink, Hazel McLellan, Miles Armstrong, Tatyana Bukharova, Zhiwei Qin, Paul R.J. Birch

**Affiliations:** 1 Horticultural College, Northeast Agricultural University, No. 59 Mucai Road, Harbin 150030, China; 2 Cell and Molecular Sciences, James Hutton Institute, Errol Road, Invergowrie, Dundee DD2 5DA, UK; 3 Division of Plant Sciences, College of Life Sciences, University of Dundee (at JHI), Errol Road, Invergowrie, Dundee DD2 5DA, UK; 4 Virus-free Seedling Research Institute of Heilongjiang Academy of Agricultural Sciences, No. 368 Xuefu Road, Harbin 150086, China

**Keywords:** effector-triggered susceptibility, oomycete, plant disease, late blight

## Abstract

Plant pathogens deliver effector proteins that alter host processes to create an environment conducive to colonization. Attention has focused on identifying the targets of effectors and how their manipulation facilitates disease. RXLR effector Pi04089 from the potato blight pathogen *Phytophthora infestans* accumulates in the host nucleus and enhances colonization when transiently expressed *in planta*. Its nuclear localization is required for enhanced *P. infestans* colonization. Pi04089 interacts in yeast and *in planta* with a putative potato K-homology (KH) RNA-binding protein, StKRBP1. Co-localization of Pi04089 and StKRBP1, and bimolecular fluorescence complementation between them, indicate they associate at nuclear speckles. StKRBP1 protein levels increased when it was co-expressed with Pi04089. Indeed, such accumulation of StKRBP1 was observed also on the first day of leaf colonization by the pathogen. Remarkably, overexpression of *StKRBP1* significantly enhances *P. infestans* infection. Mutation of the nucleotide-binding motif GxxG to GDDG in all three KH domains of StKRBP1 abolishes its interaction with Pi04089, its localization to nuclear speckles, and its increased accumulation when co-expressed with the effector. Moreover, the mutant StKRBP1 protein no longer enhances leaf colonization by *P. infestans*, implying that nucleotide binding is likely required for this activity. We thus argue that StKRBP1 can be regarded as a susceptibility factor, as its activity is beneficial to the pathogen.

## Teaser

One of the key questions in plant pathology is how the effector proteins from microbial pathogens alter host processes to promote susceptibility. We show that an effector from the potato late blight pathogen targets a host RNA-binding protein, increasing its stability. Remarkably, the RNA-binding protein acts as a susceptibility factor, enhancing disease development.

## Introduction

The first line of inducible defense in plants involves the recognition of conserved microbial molecules (pathogen-associated molecular patterns; PAMPs) or molecules generated as a result of cellular damage (DAMPs), leading to the activation of defense pathways. This can be referred to as pattern-triggered immunity (PTI) (Dou and Zhou, 2012). Host-adapted pathogens have evolved to manipulate or suppress PTI to promote susceptibility (Jones and Dangl, 2006). One of the strategies used for PTI suppression involves the secretion of effectors, which may function in the apoplastic interface between the pathogen and host or be translocated into host cells. The study of plant pathogen effectors has expanded rapidly into a recognized field. Identification of the host target proteins and functions of effectors, while still in its infancy, is revealing fascinating insights into how plant pathogens manipulate host defense pathways and processes (Win et al., 2013). Most progress to date has been made in understanding bacterial type III secreted effectors (Block and Alfano, 2011; Deslandes and Rivas, 2012; Dou and Zhou, 2012), and relatively little is understood about the targets of effectors from filamentous plant pathogens such as fungi and oomycetes. Oomycetes include pathogens of considerable economic and environmental impact, range from obligate biotrophy to necrotrophy, and deploy a variety of apoplastic and intracellular (cytoplasmic) effectors (Kamoun et al., 2014). Perhaps the best studied group of cytoplasmic oomycete effectors is the RXLR class, named after a conserved Arg-x-Leu-Arg (RXLR) motif that is required for their translocation from the pathogen to the inside of host plant cells (Whisson et al., 2007; Dou et al., 2008). A critical next step following the discovery of RXLR effectors is their use as probes to reveal the host proteins, processes, and mechanisms that are targeted to promote susceptibility.

Plant immunity involves a complex network of cross-linked signaling and regulatory processes. Regulation occurs at every level, from gene expression to protein modification and turnover. It appears that effector complements from plant pathogens have evolved to target many different levels and aspects of the defense network, and potentially other processes that facilitate pathogen development and/or nutrition (Mukhtar et al., 2011; Weßling et al., 2014). The indications are that the oomycete RXLR effector class alone targets a wide range of immune-associated processes. In the case of the potato late blight pathogen *Phytophthora infestans*, more than one mitogen-activated protein kinase (MAPK) pathway has been implicated in defense signaling from cell surface receptors that detect PAMPs, and *P. infestans* RXLR effectors specifically and redundantly target these distinct MAPK signal transduction pathways (King et al., 2014; Zheng et al., 2014). Other effectors work downstream of MAPK signaling to suppress expression of the flg22-responsive *FRK1* promoter (Zheng et al., 2014). Effector *Pi*03192 prevents the nuclear translocation, and thus presumably the normal activity, of membrane-located NAC transcription factors (McLellan et al., 2013). *P. infestans* Avrblb2 interferes with secretion of a defense protease (Bozkurt et al., 2011) and AVR3a prevents or alters the normal activity of E3 ligase CMPG1, thereby inhibiting the cell death pathway stimulated by one of its PAMPs, INF1 (Bos et al., 2010). AVR2 from *P. infestans* targets the brassinosteroid signal transduction component BSL1, which is required for recognition of this effector by the host resistance protein R2, although why AVR2 interacts with BSL1 remains unknown (Saunders et al., 2012). In addition, RXLR effectors from *Phytophthora sojae* have been shown to attenuate immunity through suppression of gene silencing (Qiao et al., 2013) and through NADH and ADP-ribose pyrophosphorylase enzyme activity (Dong et al., 2011). Moreover, a *Hyaloperonospora arabidopsidis* RXLR effector suppresses salicylic acid-mediated gene expression by targeting a subunit of the Mediator complex for proteasomal degradation (Caillaud et al., 2013). Many of these examples reveal effector-mediated suppression of immunity and/or direct inhibition of the normal activity of a target protein. To date, an oomycete effector target with activity that is beneficial to infection has not been reported.

Here, we demonstrate that candidate *P. infestans* RXLR effector PITG_04089 (Pi04089) is up-regulated during the biotrophic stage of infection, enhances *P. infestans* infection, and associates with the plant nucleus and nucleolus. Pi04089 is a member of RXLRfam5, a family of RXLR effectors that includes AvrBlb2 family members from *P. infestans* and homologs in *P. sojae*, but contains no known functional domains (Haas et al., 2009). The nuclear location of the effector is required and is sufficient for its enhancement of infection. Yeast two-hybrid (Y2H) screening, co-immunoprecipitation, and bimolecular fluorescence complementation indicate that Pi04089 interacts with a potato K-homology (KH) class putative RNA-binding protein, called StKRBP1, and that this interaction occurs in the plant nucleus. The effector interaction is abolished by mutation of all three StKRBP1 KH domains. We provide evidence that StKRBP1 protein accumulates in the presence of Pi04089 and during the early stages of *P. infestans* infection. We show that it is a positive regulator of infection, dependent on functional nucleotide-binding domains, and we thus argue that it acts as a susceptibility factor.

## Results

### RXLR Effector Pi04089 Functions in the Host Nucleus to Enhance *P. infestans* Colonization

**Figure 1:**
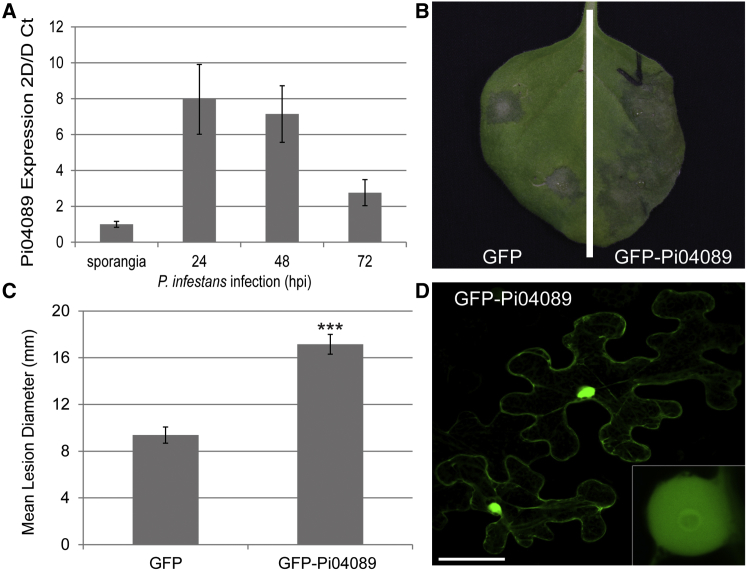
Pi04089 Contributes to *P. infestans* Virulence. **(A)***Pi04089* expression is up-regulated at 24 and 48 h post infection (hpi) of potato plants with *P. infestans*. **(B)** Increase in the area colonized by *P. infestans* following *Agrobacterium*-mediated expression of GFP-04089 in one half of a leaf compared with the expression of a GFP control. **(C)** Graph shows a significant increase (*p* < 0.001, *t* test, as indicated by asterisks) in mean diameter of *P. infestans* lesions following *Agrobacterium*-mediated expression of GFP-04089 compared with the expression of a GFP control. Error bars are SE, and the graph represents the combined data from three biological reps (*n* = 84 per construct). **(D)** Confocal projection of *N. benthamiana* leaf epidermal cells transiently expressing GFP-04089, showing that the fusion protein accumulates in the cytoplasm and nucleus. The inset is a magnified single optical section through the cell nucleus, showing that the effector fusion forms a distinct ring around the nucleolus. Scale bar represents 50 μm.

Putative RXLR effector PITG_04089 (Pi04089) was shown previously to be one of a small set of RXLR genes up-regulated at 2 days post-*P. infestans* inoculation of potato leaves in both genotype T30-4 (Haas et al., 2009; Cooke et al., 2012) and the prevalent contemporary genotype 13_A2 (Blue13) (Cooke et al., 2012). We used quantitative RT–PCR to demonstrate that Pi04089 is up-regulated also in isolate 88069 at 24 and 48 h after inoculation of susceptible potato cv. Bintje (Figure 1A). This corresponds to the early stages of the biotrophic phase of infection, which normally extends to 72 h after inoculation in this isolate (Whisson et al., 2007; Avrova et al., 2008).

To investigate whether Pi04089 acts within host cells to promote *P. infestans* infection, we transiently expressed the mature protein-coding region of Pi04089 (without signal peptide) in leaves of the model solanaceous plant *Nicotiana benthamiana*, which is a host for *P. infestans* (Bos et al., 2010; McLellan et al., 2013; King et al., 2014; Zheng et al., 2014). *Agrobacterium*-mediated transient expression of GFP-Pi04089, tagged at the N terminus with GFP, significantly enhanced *P. infestans* lesion size compared with an unfused GFP control (Figure 1B and 1C). GFP-Pi04089 expressed transiently in *N. benthamiana* leaves was stable as an intact fusion protein (Supplemental Figure 1) and predominantly located in the nucleus, forming a ring around the nucleolus, but was detected also in the cytoplasm (Figure 1D). The same pattern of localization was observed with the RXLR effector SFI3/PITG_06087/pexRD16 (Zheng et al., 2014), suggesting that this effector would make a suitable control in our experiments with Pi04089. Interestingly, whereas both Pi04089 (Figure 1) and SFI3 (Zheng et al., 2014) share similar intracellular localization patterns and each enhances *P. infestans* leaf colonization, only SFI3 was able to suppress early transcriptional changes following treatment with the bacterial PAMP flg22 (Zheng et al., 2014), indicating that the two effectors are unlikely to share a similar function.

**Figure 2:**
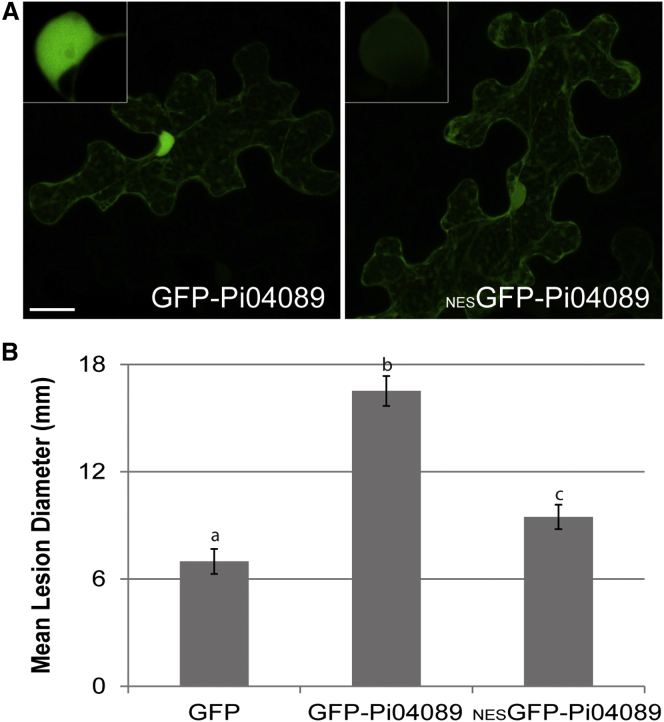
Nuclear Localization of Pi04089 Is Necessary to Promote *P. infestans* Colonization. **(A)** Images are confocal projections of *N. benthamiana* leaf epidermal cells transiently expressing the GFP-Pi04089 and the modified _NES_GFP-Pi04089, showing the reduction in nuclear fluorescence resulting from the addition of the nuclear export signal. Insets are magnified single optical sections of the cell nuclei, revealing that fluorescence associated with the nucleus in the stacked projection is primarily in cytoplasm surrounding the nucleus. Scale bar represents 20 μm. **(B)** Graph shows a significant decrease (*p* < 0.001 by analysis of variance [ANOVA, as indicated by lowercase letters]) in mean diameter of *P. infestans* lesions following *Agrobacterium*-mediated expression of _NES_GFP-Pi04089 compared with the expression of GFP-Pi04089. Error bars are SE, and the graph represents the combined data from three biological reps (*n* = 142 per construct).

To determine whether the nuclear location of Pi04089 is important to the function of the effector, a nuclear export signal (NES) was added to the N terminus of the GFP fusion form (_NES_GFP-Pi04089). As anticipated, the _NES_GFP-Pi04089 showed greatly reduced fluorescence in the plant cell nucleus, and correspondingly increased cytoplasmic fluorescence (Figure 2A). Transient expression of _NES_GFP-Pi04089 did not support enhanced leaf colonization of *P. infestans* compared with the unmodified GFP-Pi04089 (Figure 2B). In contrast, accumulating the effector fusion in the nucleus with the addition of a nuclear localization signal (NLS) increased the proportion of the GFP fluorescence in the nucleus in expressing cells, with little cytoplasmic background remaining, but made no significant impact on the ability of the effector fusion to increase *P. infestans* leaf colonization (Supplemental Figure 2). The modified (NES or NLS) GFP fusion proteins were intact and stable *in planta* (Supplemental Figure 2).

### Pi04089 Interacts with a Putative RNA-Binding Protein with Three KH Domains

**Figure 3:**
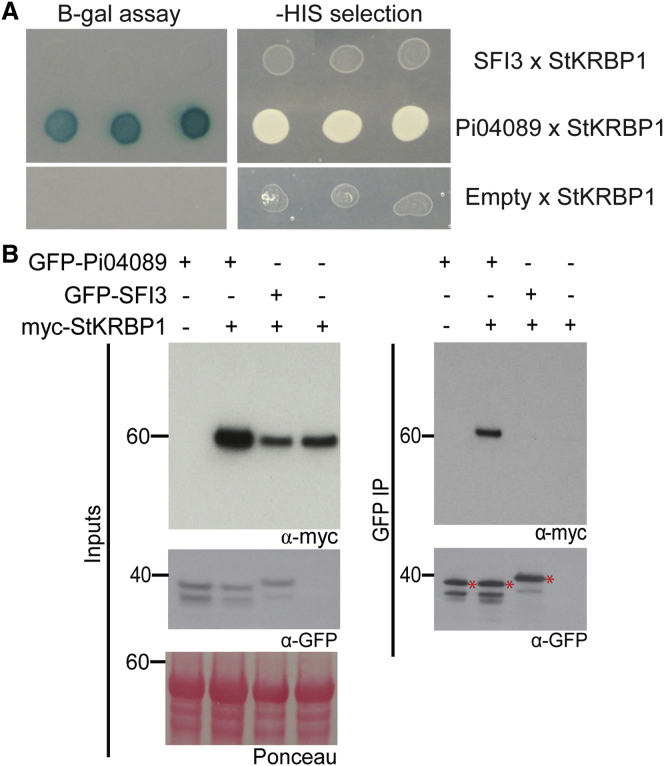
Pi04089 Interacts with a Potato Predicted KH RNA-Binding Protein in Y2H and *In Planta*. **(A)** Yeasts co-expressing the StKRBP1 with Pi04089 grow on -histidine (-HIS) medium and have β-galactosidase (B-gal) activity, whereas yeasts co-expressing SFI3 or empty vector (Empty) do not. **(B)** Co-immunoprecipitation from leaf extracts using GFP-trap (GFP IP) confirmed that StKRBP1 specifically interacted with Pi04089 and not with SFI3. Expression of constructs is indicated by plus signs. Effector protein fusion bands are indicated by asterisks. Protein size markers are indicated in kDa, and protein loading is indicated by Ponceau stain.

To search for possible host target proteins of Pi04089, a Y2H library created from infected potato cDNA (Bos et al., 2010) was screened with a GAL4 DNA-binding domain:Pi04089 fusion construct (“bait”). Approximately 2.82 million yeast co-transformants were screened, and 15 yeast colonies were recovered from selection plates that contained GAL4 activation domain (“prey”) fusions to sequences corresponding to potato PGSC0003DMT400066837. This gene encodes a protein with sequence features conserved among the KH class of RNA-binding proteins (Supplemental Figure 3). One of the prey fusions predicted to encode the full-length protein sequence was tested against the bait vectors containing Pi04089 to confirm the interaction, using effector SFI3 as a control. Interaction in yeast was detected specifically between *Solanum tuberosum* (St)KRBP1 and Pi04089, and not between StKRBP1 and SFI3 (Figure 3A).

A cMyc tag was fused to the amino terminus of the StKRBP1 protein, and this fusion was transiently expressed in *N. benthamiana* leaves alone and with either GFP-Pi04089 or GFP-SFI3 using *Agrobacterium*. Protein extracts from these plants were used for co-immunoprecipitation. The cMyc-tagged StKRBP1 was only co-immunoprecipitated when co-expressed with GFP-Pi04089 (Figure 3B), confirming specific *in planta* association with this effector.

### Pi04089–StKRBP1 Interaction Occurs at Distinct Subnuclear Locations

**Figure 4:**
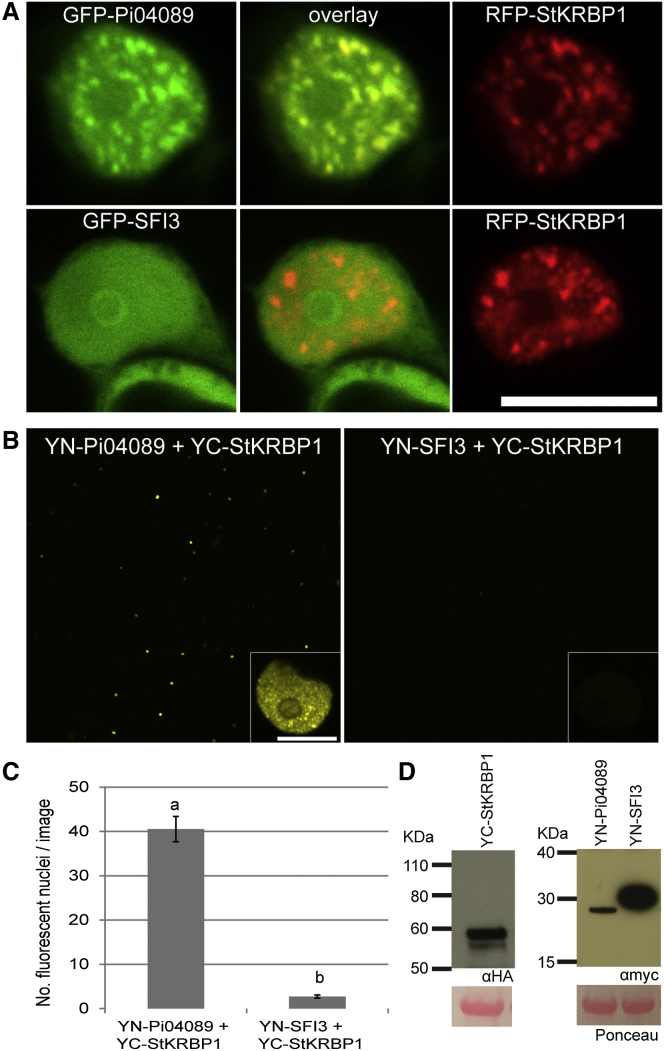
StKRBP1 Specifically Relocalizes Pi04089 to Nuclear Speckles. **(A)** Single optical sections of *N. benthamiana* leaf epidermal cell nuclei transiently co-expressing the GFP-Pi04089 or GFP-SFI3 with RFP-StKRBP1, showing that GFP-Pi04089 is relocated to nuclear speckles, and the nucleolar ring is no longer observed while the localization of GFP-SFI3 remains unaffected. Scale bar represents 10 μm. **(B)** Low-magnification images collected with identical imaging parameters and a 10× lens of *N. benthamiana* leaves, showing that bimolecular fluorescence between YN-Pi04089 and YC-StKRBP1 is more frequently observed than with the YN-SFI3 control and is located in the nuclei of expressing cells. Insets are single optical sections of nuclei, showing that the fluorescence between YN-Pi04089 and YC-StKRBP1 is located in the nuclear speckles. Inset for the YN-SFI3 plus YC-StKRBP1 control combination indicates the level of background non-specific fluorescence observed in a small number of cells. **(C)** Graph shows the average number of nuclei observable per field of view using the 10× lens and identical settings for each of the combinations. Significantly more nuclei are observed for YN-Pi04089 and YC-StKRBP1 compared with YN-SFI3 and YC-StKRBP1 (*p* < 0.001, *t* test, as indicated by lowercase letters). Error bars are SE, and the graph represents the data from one biological rep (*n* = 11 fields of view per construct). **(D)** Immunoblots indicate that each of the constructs used for bimolecular fluorescence experiments are stable and of the expected size.

StKRBP1 was fused to mRFP or GFP and the fusion was transiently expressed in *N. benthamiana* leaves. In cells with moderate levels of expression, the fluorescence was located in the nucleus and formed a pattern of random speckles (Supplemental Figure 4A). When co-expressed with GFP-Pi04089, the effector fusion co-located with the RFP-StKRBP1 and was no longer visible as a ring around the nucleolus (Figure 4A). In contrast, GFP-SFI3 remained as smooth nucleoplasmic fluorescence, retaining the ring around the nucleolus, when co-expressed with RFP-StKRBP1 (Figure 4A).

A bimolecular fluorescence complementation (BiFC) assay was performed with the YFP amino-terminal fragment (YN) fused to the effectors and the YFP carboxy-terminal fragment (YC) fused to StKRBP1. Low agrobacterial concentrations (OD_600_ 0.002) were used, and the infiltrated leaves were observed approximately 1.5 days after agroinfiltration to minimize the non-specific fluorescence that often occurs with split YFP constructs when they are overexpressed (Boevink et al., 2014). YFP fluorescence generated by co-expression of YN-04089 with YC-StKRBP1 was restricted to the nucleus, and especially emphasized the nuclear speckles (Figure 4B). In contrast, little fluorescence was observed following co-expression of YN-SFI3 with YC-StKRBP1 (Figure 4B). This was evident when the numbers of visibly fluorescent nuclei in low-magnification images collected with identical microscope settings were counted, indicating a significantly higher number of fluorescent nuclei with the Pi04089 fusion than with the SFI3 control (Figure 4C). Each of these constructs was stable *in planta* (Figure 4D).

### Overexpression of the StKRBP1 Enhances *P. infestans* Growth

To investigate whether KRBP1 was likely to act as a positive regulator of immunity, we silenced it using virus-induced gene silencing (VIGS). Two independent constructs were generated in a tobacco rattle virus (TRV) vector for VIGS of the *N. benthamiana* ortholog *NbKRBP1* (Supplemental Figure 5A). Plants were inoculated with the viruses carrying the silencing constructs, and the levels of silencing were analyzed after 21 days. Both VIGS constructs significantly reduced *Nb*KRBP1 transcript levels compared with a TRV::GFP control construct (Supplemental Figure 5B). The infectivity of *P. infestans* inoculated onto silenced leaves, as measured by the number of lesions that developed and the number of sporangia formed, was not significantly affected by the reduction in the *NbKRBP1* transcript (Supplemental Figure 5C and 5D).

**Figure 5:**
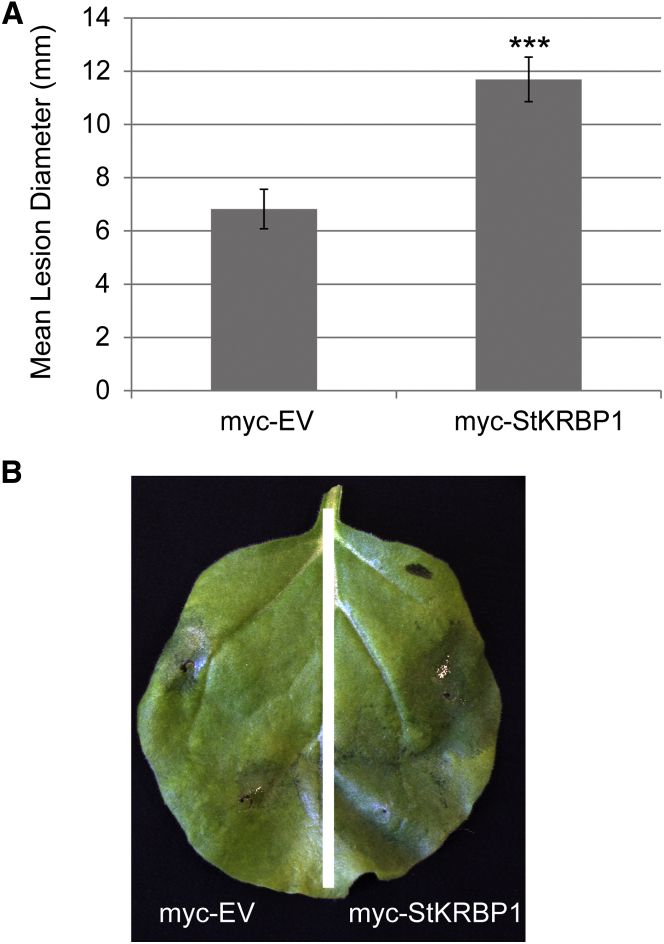
Overexpression of StKRBP1 Promotes *P. infestans* Colonization. **(A)** Graph shows a significant increase (*p* < 0.001, *t* test, as indicated by asterisks) in mean diameter of *P. infestans* lesions following *Agrobacterium*-mediated expression of myc-StKRBP1 compared with the expression of the empty vector. Error bars are SE, and the graph represents the combined data from three biological reps (*n* = 194 per construct). **(B)** An example leaf showing a larger water-soaked lesion on the side infiltrated with myc-StKRBP1.

To further investigate the effect of the StKRBP1 on *P. infestans* infection, the cMyc-tagged protein, or the empty cMyc vector as a control, was transiently overexpressed in leaves and the leaves were subsequently inoculated with *P. infestans* strain 88069. After 7 days the lesion diameters were measured. Remarkably, overexpression of the StKRBP1 caused a significant increase (*p* < 0.01 by analysis of variance) in infection lesion diameter (Figure 5).

### StKRBP1 Is Stabilized by Pi04089 and during the Early Stages of *P. infestans* Infection

**Figure 6:**
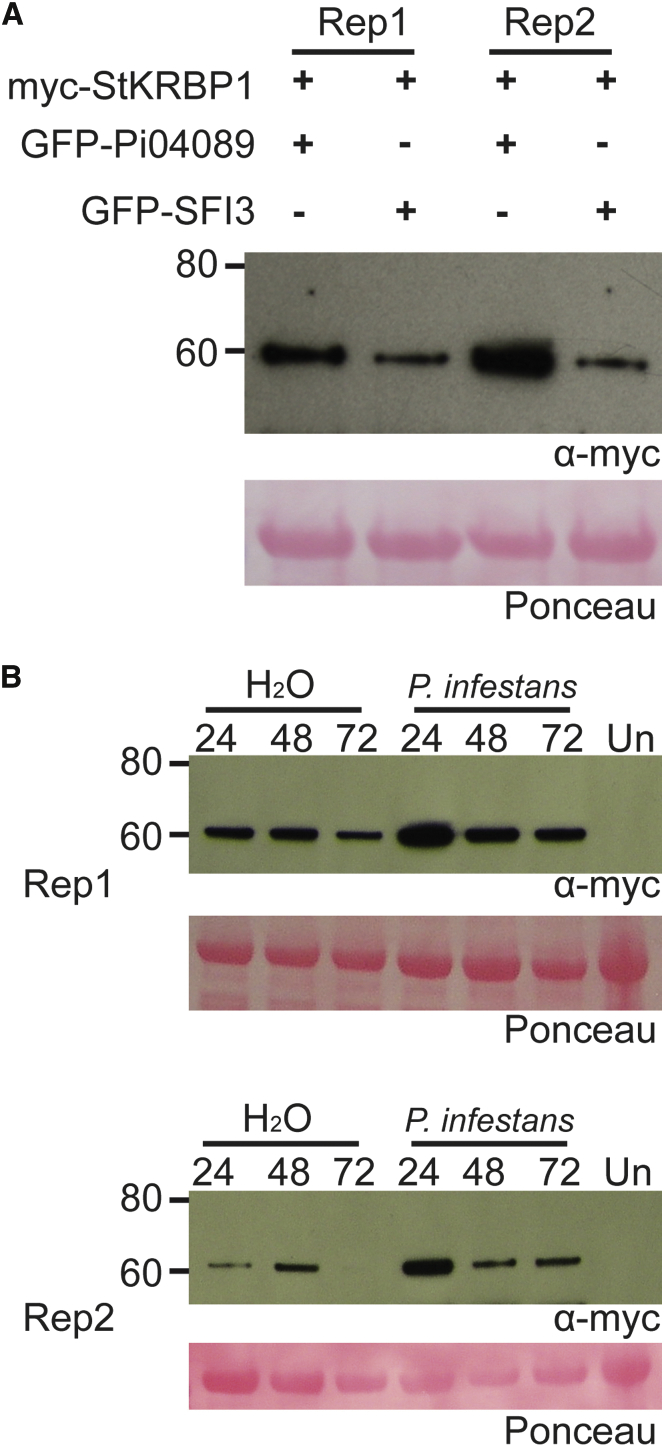
StKRBP1 Is Stabilized by Pi04089 and *P. infestans* Infection. **(A)** Immunoblot showing an increased signal from myc-StKRBP1 in the presence of GFP-Pi04089 and not with GFP-SFI3. **(B)** Immunoblots from two biological replicates showing that the myc-StKRBP1 is more stable following infection with *P. infestans* compared with a water-inoculated control. 24, 48, and 72 indicate hours post treatment with water or *P. infestans*, Un, a non-agro-infiltrated control.

In co-immunoprecipitation assays (e.g. Figure 3), there was detectably more of the cMyc-StKRBP1 protein in the input material from leaves co-expressing GFP-Pi04089 than in the samples from either co-expression with GFP-SFI3 or expression of the cMyc-StKRBP1 alone. To determine whether this was a particular feature of co-expression with GFP-Pi04089, further Western blots were performed. There was consistently more of the cMyc-StKRBP1 in the presence of GFP-Pi04089 than with GFP-SFI3, suggesting the effector promotes the observed increase in protein abundance (Figure 6A). Given that *Pi04089* transcripts predominantly accumulate within the first day of *P. infestans* infection (Figure 1A), we assessed whether the StKRBP1 was stabilized during these initial stages of infection. Leaves transiently expressing cMyc-StKRBP1 were either mock-inoculated with water alone or with water containing *P. infestans* zoospores. There was consistently more cMyc-StKRBP1 protein at 24 h after *P. infestans* inoculation compared with either the water control or later time points after inoculation of the pathogen (Figure 6B), indicating that transient Pi04089 expression and *P. infestans* infection both promote StKRBP1 protein accumulation.

### A Mutated StKRBP1 Does Not Interact with Pi04089 or Promote *P. infestans* Infection

Mutation of the conserved GxxG motif in KH RBPs to GDDG has been shown to prevent nucleotide binding in KH domain-containing RNA-binding proteins (Hollingworth et al., 2012), without altering protein stability. To determine whether such a mutant would still interact with the effector and promote *P. infestans* leaf colonization, all three GxxG motifs in StKRBP1 were mutated to GDDG (to create StKRBP1mut). The positions of the mutations are indicated in Supplemental Figure 3.

**Figure 7:**
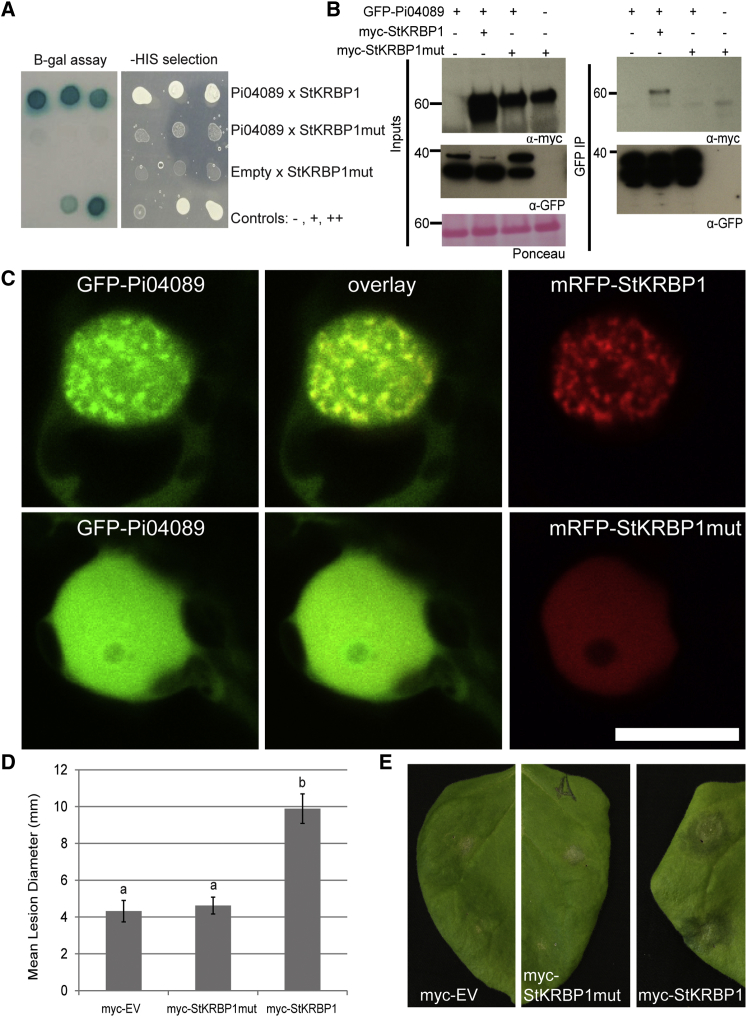
A KRBP1 Mutant No Longer Interacts with Pi04089 and Fails to Promote *P. infestans* Colonization. **(A)** Yeasts co-expressing the StKRBP1 with Pi04089 grow on -histidine (-HIS) medium and have β-galactosidase (B-gal) activity, whereas yeasts co-expressing StKRBP1 mutant (StKRBP1mut) do not. The control combination of StKRBP1mut with the empty prey vector indicates that it does not autoactivate. Controls show no (-), weak (+), and strong (++) interactions. **(B)** Co-immunoprecipitation confirmed that Pi04089 specifically interacted with StKRBP1 and not with the mutated protein. **(C)** Single optical sections of nuclei of cells co-expressing GFP-Pi04089 with either the wild-type StKRBP1 or the mutated form, showing that GFP-Pi04089 is only removed from the nucleolar periphery and relocated to speckles by the wild-type, and also that the mutated StKRBP1 does not locate to nuclear speckles. Scale bar represents 10 μm. **(D)** Graph shows that the mean diameter of *P. infestans* lesions following *Agrobacterium*-mediated expression of myc-StKRBP1 mutant was significantly decreased compared with the expression of the wild-type myc-StKRBP1 and did not significantly differ from the empty vector (myc-EV) control (*p* < 0.001, ANOVA, as indicated by lowercase letters). Error bars are SE, and the graph represents the combined data from three biological reps (*n* = 72 per construct). **(E)** Example infection sites on *N. benthamiana* leaves.

The mutated form no longer interacted with Pi04089 either in yeast using Y2H (Figure 7A) or *in planta* using co-immunoprecipitation (Figure 7B). Co-expression with the effector did not promote increased StKRBP1mut protein accumulation as it did with the wild-type form. The StKRBP1mut was tagged with mRFP and GFP, and was found to locate to the nucleoplasm but not to form the nuclear speckles seen with the wild-type form (Figure 7C and Supplemental Figure 6). When co-expressed with GFP-04089 the mRFP-StKRBP1mut did not alter the location of the effector fusion (Figure 7C). Critically, whereas cMyc-StKRBP1 wild-type enhanced *P. infestans* leaf colonization, transient expression of cMyc-tagged StKRBP1mut did not result in increased pathogen colonization compared with the empty vector control (Figure 7D and 7E).

## Discussion

The RXLR effector Pi04089 acts in the nucleus to promote *P. infestans* colonization, and interacts in yeast and *in planta* with StKRBP1, a predicted KH RBP from *S. tuberosum*. Co-localization of fluorescently tagged Pi04089 and StKRBP1, and BiFC, indicate that these proteins associate at speckles within the nucleus. Co-expression of Pi04089 and StKRBP1 results in increased protein accumulation of the latter. Indeed, increased protein accumulation of StKRBP1 is also seen during the earliest stages of leaf colonization by the pathogen. Remarkably, overexpression of either Pi04089 or StKRBP1 enhances *P. infestans* leaf colonization, implying that the effector target functions in a way that is beneficial to infection, and that the effector does not, therefore, inhibit the activity of StKRBP1. Mutation of the putative nucleotide-binding sites in all three of the KH domains of StKRBP1 abolished its interaction with Pi04089 in yeast and *in planta*, prevented its localization at nuclear speckles, and attenuated its increased accumulation when co-expressed with the effector. Moreover, the mutant StKRBP1 protein no longer supported enhanced leaf colonization by *P. infestans*, indicating that nucleotide binding may be required for this activity. Each of these observations is discussed.

Pi04089 transient expression inside plant cells provided a significant enhancement to *P. infestans* colonization, suggesting that the activity of the effector is beneficial to infection. The effector was observed to accumulate in the cytoplasm and nucleus, forming a ring around the nucleolus. The beneficial activity of the effector was lost when it was exported out of the nucleus with an NES, suggesting that the nucleus is an important site of Pi04089 action. Importantly, when Pi04089 was focused in the nucleus with an NLS the beneficial activity of the effector was not diminished, suggesting that a cytoplasmic phase was not critical for Pi04089 function. We thus conclude that Pi04089 is a nuclear effector. This is further supported by the identification of a host putative KH RBP, which shows a distinctive subnuclear localization, as an interactor of Pi04089 in yeast, using Y2H, and *in planta*, using co-immunoprecipitation and BiFC. Critically, both co-localization of effector and candidate target, and the BiFC between them, revealed that they are closely associated with each other at nuclear speckles, further emphasizing the plant nucleus as the site of Pi04089 activity.

Research on plant immunity has revealed many proteins and processes involved in pathogen perception, signal transduction, and changes at both the transcriptional and post-translational level. Less attention has been given to post-transcriptional regulation of immunity, although it is apparent that pre-mRNA splicing (e.g. Dinesh-Kumar and Baker, 2000), and polyadenylation (Lyons et al., 2013), stability, silencing, and transport of mRNA have all been shown to be differential in response to pathogen challenge (Staiger et al., 2012). All of these processes involve the intimate activity of RBPs. RBPs are defined by RNA-binding domains, such as the RNA recognition motif (RRM) and KH domain. A number of RRM-containing putative RBPs have been implicated as regulators of plant immunity. RBP-DR1, containing three RRMs, contributes to basal defense to *Pseudomonas syringae*. Its overexpression leads to enhanced levels of salicylic acid (Qi et al., 2010), but its binding to nucleotides has not yet been characterized. In contrast, the RRM-containing flowering regulator FPA acts as a negative regulator of immunity. FPA inhibits the PTI-mediated production of an alternatively polyadenylated form of the transcriptional repressor ERF4 (Lyons et al., 2013). A further RRM-containing RBP, GRP7, while positively regulating floral transition (Streitner et al., 2008), also acts as a positive regulator of immunity (Fu et al., 2007). GRP7 is directly targeted by the *P. syringae* type III secreted effector HopU1, which possesses mono-ADP ribosyltransferase activity (Fu et al., 2007). HopU1 mono-ADP ribosylates arginine 49 in the RRM, and thus prevents GRP7 binding to RNA (Jeong et al., 2011). More recently, it has been shown that GRP7 interacts with transcripts from the receptor gene *FLS2* and that this is prevented by HopU1, resulting in reduced FLS2 protein levels during infection (Nicaise et al., 2013).

KH RBPs are found in prokaryotes and eukaryotes, with eukaryotic RBPs usually possessing more than one KH domain. They are involved in a range of activities, including the coordination of mRNA synthesis and metabolism, and facilitation of mRNA transport and translational control (Valverde et al., 2008). Few KH RBPs have been characterized in plants. Antagonistic roles have been proposed for two KH RBPs involved in regulating flowering time: FLK and PEPPER. The former promotes flowering by reducing transcript abundance of the flowering repressor FLC, whereas PEPPER delays flowering by increasing the abundance of FLC transcripts (Ripoll et al., 2009). In addition, a KH RBP called HOS5 is involved in tolerance to abiotic stress and is involved in pre-mRNA splicing. Hos5 associates with splicing factors in nuclear speckles (Chen et al., 2013). To our knowledge, KH domain RBPs have yet to be implicated in regulating plant immunity.

StKRBP1 and Pi04089 co-localize to subnuclear structures reminiscent of nuclear speckles. Nuclear speckles are strongly associated with pre-mRNA splicing factors and are often localized with, or adjacent to, sites of high transcriptional activity (Spector and Lomond, 2011). Differential or alternative splicing has been shown to be a major post-transcriptional regulator of stress responses, especially in plants (Staiger and Brown, 2013). A number of plant splicing factors have been localized to nuclear speckles and co-localization studies have indicated that different splicing factors can be associated with distinct speckles (Reddy et al., 2012). It will thus be interesting to investigate whether StKRBP1, like the KH RBP Hos5 referred to above (Chen et al., 2013), co-localizes with splicing factors, potentially implicating its activity with pre-mRNA processing.

The KH domain has largely been associated with binding to RNA. However, it can also be associated with binding single-stranded (ss) DNA (Valverde et al., 2008). While it will be necessary for future detailed experiments to investigate which specific class of nucleotides StKRBP1 binds to, its association with nuclear speckles likely implicates RNA; such speckles are otherwise referred to as interchromatin granules (ICGs), which are often rich in RNA and lack DNA (Spector and Lomond, 2011). Whether bound to RNA or ssDNA, the conserved GxxG motif within the KH domain is required for direct nucleotide binding and is the critical component of a binding pocket that associates with specific stretches of four nucleotides. Further nucleotide-binding specificity is provided in eukaryotes by the presence of two or more KH domains, each associated with four nucleotides. Mutation of the GxxG motif to GDDG has been shown to abolish nucleotide binding (Hollingworth et al., 2012). We thus mutated the GxxG motif in all three of the KH domains in StKRBP1 to form StKRBP1mut. StKRBP1mut no longer localized to nuclear speckles, potentially consistent with a loss of nucleotide binding. Interestingly, the interaction with the effector Pi04089 was also abolished. Correspondingly, Pi04089 was not localized to nuclear speckles when co-expressed with StKRBP1mut. These data suggest three possibilities for Pi04089 binding to StKRBP1: (1) it could be a direct protein–protein interaction with the KH domain itself (which is altered in conformation by the GDDG mutation); (2) it binds with a KH domain–nucleotide complex; or (3) Pi04089 interacts with other facets of StKRBP1 that are altered in conformation as a result of nucleotide binding. The interaction between StKRBP1 and Pi04089 using a Y2H assay supports the former of these possibilities. The alternatives require that StKRBP1 is able to associate with RNA or ssDNA also in yeast cells, which, given the potential specificity to plant nucleotide sequences, is unlikely. Interestingly, it is emerging that the KH domains in plant KH RBPs may also act as protein–protein interaction domains (Chen et al., 2013; Jeong et al., 2013; Jiang et al., 2013), suggesting that conformational changes made by the GDDG mutation may result in changes to both nucleotide and protein interactions. Future detailed work is needed to characterize the nucleotide-binding specificity and host protein binding partners of StKRBP1 to decipher its regulatory role in plants.

Co-expression of Pi04089 with StKRBP1 results in increased protein levels of the latter. Interestingly, such increased StKRBP1 protein accumulation is also seen in the first 24 h after inoculation of *P. infestans* spores onto leaves (Figure 6), coinciding with the peak transcript accumulation of *Pi04089* (Figure 1). Overexpression of either StKRBP1 (Figures 5 and 7) or Pi04089 (Figures 1 and 2) each independently promote enhanced *P. infestans* leaf colonization. Critically, StKRBP1mut, in which all three KH RNA-binding domains are disabled, no longer interacts with Pi04089 or enhances *P. infestans* colonization (Figure 7). Taken together, these data indicate that Pi04089 is unlikely to inhibit StKRBP1 activity, as this activity is beneficial to the pathogen. Moreover, as StKRBP1 overexpression enhances infection, it can thus be regarded as a susceptibility factor.

The notion of susceptibility (S) factors covers a broad range of host activities that support pathogen infection, from physical alterations to, for example, cell wall composition or stomatal opening, to proteins that suppress or antagonize immunity, to those that provide nutrition to support pathogen growth (reviewed in van Schie and Takken, 2014). Many have been designated as S factors because of the observed detrimental effects on pathogen infection when they are mutated, and/or positive effects when they are overexpressed. However, there are relatively few examples whereby the contribution of a host gene or protein to susceptibility is consequent upon direct effector activity. Examples include: *Xanthomonas* TAL effectors that have been shown to directly induce expression of *SWEET* genes, which contribute to sugar efflux and, thus, pathogen nutrition (e.g. Chen et al., 2010); the *P. syringae* type III secreted effector AvrB that mediates the phosphorylation and, thus, activation of MPK4, which is a suppressor of PTI (Cui et al., 2010); and nematode effectors that bind to pectin methylesterases that have been shown to act as S factors (Hewezi et al., 2008).

The enhancement of *P. infestans* colonization when StKRBP1 is overexpressed is consistent with it being regarded as an S factor. Silencing of *NbKRBP1*, however, did not significantly reduce *P. infestans* infection as might be anticipated. Nevertheless, silencing by VIGS only knocked down the transcript levels of *NbKRBP1* by approximately 65% (Supplemental Figure 5). There may remain sufficient expression for NbKRBP1 to contribute to infection, especially as VIGS is notoriously “patchy,” with some leaf areas more efficiently silenced than others, and observing a reduction in pathogen performance is thus perhaps more challenging than an increase in susceptibility. An alternative interpretation of our observations in this work relates to the increase in stability of StKRBP1 during the early stages of infection, and when co-expressed with Pi04089, offering the possibility that the effector modifies or (re)focuses StKRBP1 activity. The future challenges will be to define the precise role of StKRBP1 and its impact on immunity, and how the effector Pi04089 may facilitate, modify, or enhance its activity to promote late blight disease.

## Methods

### Vector Construction

*P. infestans* putative RXLR effector genes PITG_04089 and PITG_06087 (SFI3) were cloned without signal peptides from gDNA of isolate T30-4 in a two-step PCR to add flanking attB sites to the coding sequences. The cloning primers are shown in Supplemental Table 1. The *S.* *tuberosum* KH domain RNA-binding protein (StKRBP1) coding sequence was amplified from a suitable GAL4 AD domain fusion construct identified in the initial Y2H screen using the same strategy (primer sequences are shown in Supplemental Table 1).

The PCR products were recombined into pDONR201 or pDONR/Zeo (Invitrogen) to generate entry clones using the Gateway recombination cloning technology (Invitrogen). The effector entry clones were recombined with pDEST32 (for Y2H; Invitrogen), pB7WGF2 (for N-terminal EGFP fusion; Karimi et al., 2002), and, for BiFC, into pCL112 (for N-terminal YN fusion; Bos et al., 2010). Modified forms of pB7WGF2 with either an NES signal derived from PKI (amino acid sequence LALKLAGLDIN; Wen et al., 1995) or an NLS signal derived from SV40 T antigen (amino acid sequence PKKKRKV; Kalderon et al., 1984) added to the N terminus of the GFP were created. The effector entry clones were also recombined with these. The KH RBP entry clone was recombined with pGWB18 (for N-terminal tagging with the cMyc epitope), pGWB461 (for N-terminal tagRFP fusion; Nakagawa et al., 2007), and pCL113 (for N-terminal YC fusion). These vectors are designed for 35S promoter-driven gene expression.

Generation of the KH RBP mutant sequence involved conversion of the two central residues in each of the three conserved GxxG motifs to aspartates; resulting in GDDG, which was found to abolish RNA binding in a number of KH domain-containing RNA-binding proteins (Hollingworth et al., 2012). The construct was synthesized by Genscript. The entry clone containing the mutated form of the KH RBP (KH RBPmut) was recombined with pK7WGR2 (for N-terminal mRFP fusion; Karimi et al., 2005), pGWB18 (for N-terminal c-Myc tagging), and pDEST22 to generate GAL4 activation domain fusions for Y2H.

### Gene Expression Assay

RNA was extracted using a Qiagen RNeasy Kit with on-the-column DNA digestion steps according to the manufacturer's instructions. First-strand cDNA was synthesized from 2 μg of RNA using Superscript II RNase HReverse Transcriptase (Invitrogen) according to the manufacturer's instructions. Real-time quantitative RT–PCR reactions were performed using Power SYBR Green (Applied Biosystems) and run on a Chromo4 thermal cycler (MJ Research, UK) using Opticon Monitor 3 software. Primer pairs were designed outside the region of cDNA targeted for silencing following the manufacturer's guidelines. Primer sequences are listed in Supplemental Table 1. Detection of real-time RT–PCR products, calculations, and statistical analysis were performed as previously described (McLellan et al., 2013).

### Plant Production and Maintenance

*N. benthamiana* plants were grown with a 16-h day at 22°C and an 8-h night at 18°C. Supplementary light was provided when the ambient light dropped below 200 W/m^2^ and shading when it was above 450 W/m^2^. Plants used were approximately 5 weeks old.

### Agroinfiltration and Infection Assays

*Agrobacterium tumefaciens* strain AGL1 transformed with vector constructs were grown overnight in YEB medium containing selective antibiotics at 28°C, pelleted, resuspended in infiltration buffer (10 mM MES, 10 mM MgCl_2_ and 200 μM acetosyringone), and adjusted to the required OD_600_ before infiltration into *N. benthamiana* leaves (generally 0.005 to 0.01 for imaging purposes, 0.002 for BiFC). For co-expression, agrobacterial cultures carrying the appropriate vector constructs were mixed prior to infiltration.

*P. infestans* strain 88069 was used for plant infection and was cultured on Rye Agar at 19°C for 2 weeks. Plates were flooded with 5 ml of H_2_O and scraped with a glass rod to release sporangia. The resulting solution was collected in a Falcon tube, and sporangia numbers were counted using a hemocytometer and adjusted to 15 000 sporangia/ml; 10-μl droplets were inoculated onto the abaxial side of detached *N. benthamiana* leaves stored on moist tissue in sealed boxes. For VIGSed plants the number of inoculated lesions sporulating at 7 days post inoculation (dpi) were counted and expressed as a percentage increase in sporulating lesions compared with the GFP control plants. Sporangia counts were performed on 10-dpi leaves from VIGSed plants that had been immersed in 5 ml of H_2_O and vortexed to release sporangia. A hemocytometer was used to count the number of sporangia recovered from each leaf and was expressed as sporangia/ml. *A. tumefaciens* transient assays (ATTA) in combination with *P. infestans* infection were carried out as described by McLellan et al. (2013). In brief, *Agrobacterium* cultures were resuspended in agroinfiltration medium at a final concentration of OD_600_ = 0.1 and used for transient expression *in planta* by agroinfiltration. After 1 day, each infiltration site was inoculated with 10 μl of zoospores from *P. infestans* isolate 88069 at 15 000 sporangia/ml.

### Confocal Imaging

*N. benthamiana* leaf cells were imaged at 2 dpi using a Leica TCS SP2 AOBS, Zeiss 710, or Nikon A1R confocal microscope with Leica HCX PL APO lbd BL 63×/1.20 W and L 40×/0.8, Zeiss PL APO 40×/1.0, or Nikon 60×/water dipping objectives. GFP was excited by the 488-nm line of an argon laser, and emissions were detected between 500 and 530 nm. tagRFP and mRFP were excited with a 561-nm line from a diode laser, and their emissions were collected between 580 and 610 nm or 600 and 630 nm, respectively. The pinhole was set to 1 airy unit for the longest-wavelength fluorophore. Single optical section images and z-stacks were collected from leaf cells expressing low levels of the protein fusions to minimize the potential artifacts of ectopic protein expression. Images were projected and processed using the Leica LCS, Zen 2010, or NIS-Elements software packages. Subsequent image processing for figure generation was conducted with Adobe Photoshop CS2 and Adobe Illustrator.

### Yeast Two-Hybrid Analysis

A Y2H screen with pDEST32-Pi04089 was performed as described by McLellan et al. (2013) using the Invitrogen ProQuest system. The full-length coding sequence of the candidate interacting prey sequence was cloned and retested with pDest32-Pi04089 and pDEST32-Pi06087 as a control to rule out the possibility that the observed reporter gene activation had resulted from interactions between the prey and the DNA-binding domain of the bait construct or DNA-binding activity of the prey itself.

### Co-Immunoprecipitation

*A. tumefaciens* strain GV3101 transformants containing fusion protein constructs were grown overnight in YEB medium containing selective antibiotics at 28°C, pelleted, resuspended in infiltration buffer (10 mM MES, 10 mM MgCl_2_, and 200 μM acetosyringone) and adjusted to an OD_600_ of 1.0 before infiltration into *N. benthamiana* leaves. Samples were taken 48 h after infiltration and proteins extracted. GFP-tagged Pi04089/SFI3 fusions were immunoprecipitated using GFP-Trap-M magnetic beads (Chromotek GmbH). The resulting samples were separated by PAGE and Western blotted. Immunoprecipitated GFP fusions and co-immunoprecipitated c-Myc fusions were detected using appropriate antisera (Santa Cruz Biotechnology, UK).

### Virus-Induced Gene Silencing

VIGS constructs were made by cloning 250-bp PCR fragments of *NbKRBP1* from *N. benthamiana* cDNA and cloning into pBinary TRV vectors (Liu et al., 2002) between *Hpa*I and *Eco*RI sites in the antisense orientation. A TRV construct expressing GFP described previously (McLellan et al., 2013) was used as a control. Primer sequences are shown in Supplemental Table 1. The two largest leaves of four-leaf stage *N. benthamiana* plants were pressure infiltrated with LBA4404 *A. tumefaciens* strains containing a mixture of RNA1 and each KH RBP VIGS construct or the GFP control at OD_600_ = 0.5 each. Plants were used for assays or to check gene silencing levels by quantitative RT–PCR 2–3 weeks later. VIGS *P. infestans* infection assays were performed as previously described (McLellan et al., 2013).

## Funding

We are grateful for financial support from the Biotechnology and Biological Sciences Research Council (BBSRC) (grants BB/G015244/1, BB/K018183/1, BB/L026880/1), and the Scottish Government Rural and Environment Science and Analytical Services Division (RESAS). X.W. was supported by funding from the China Scholarship Council (CSC).
