# Supplementary material for: A Host KH RNA-Binding Protein Is a Susceptibility Factor Targeted by an RXLR Effector to Promote Late Blight Disease
Source: Mol Plant. 2015 Sep 7;8(9):1385–95. doi: 10.1016/j.molp.2015.04.012 (PMC4560694; doi:10.1016/j.molp.2015.04.012)
Supplement: Document S1. Supplemental Figures 1–6 and Supplemental Table 1 [file mmc1.docx]

**A host KH RNA binding protein is a susceptibility factor targeted by an RXLR effector to promote late blight disease**

**Xiaodan Wang^1,2,3,4§^, Petra Boevink^2,§^, Hazel McLellan^3§^, Miles Armstrong^3^, Tatyana Bukharova^3^, Zhiwei Qin^1,^*, Paul R J Birch^2,3,^***

**Supplementary Data**

**
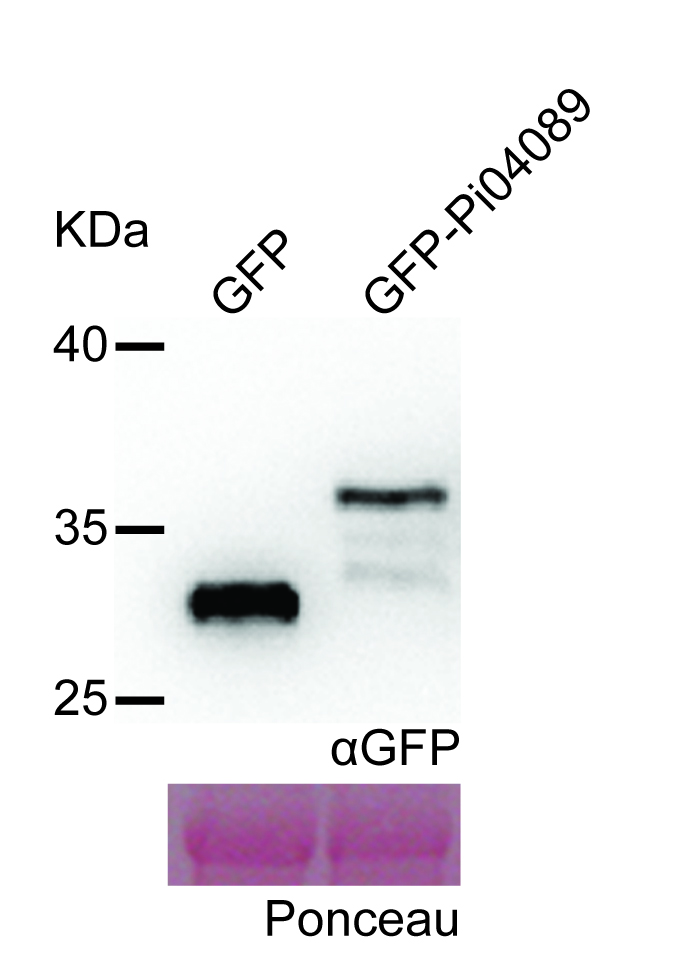
**

**Figure S1. GFP-Pi04089 Stability.** Immunoblot of the GFP-Pi04089 fusion protein (expected size of the fusion is 38 kDa) extracted from transiently expressing leaves compared to GFP extracted from a transgenic *N. benthamiana* line expressing ER-targeted GFP. The fusion is largely intact.

**
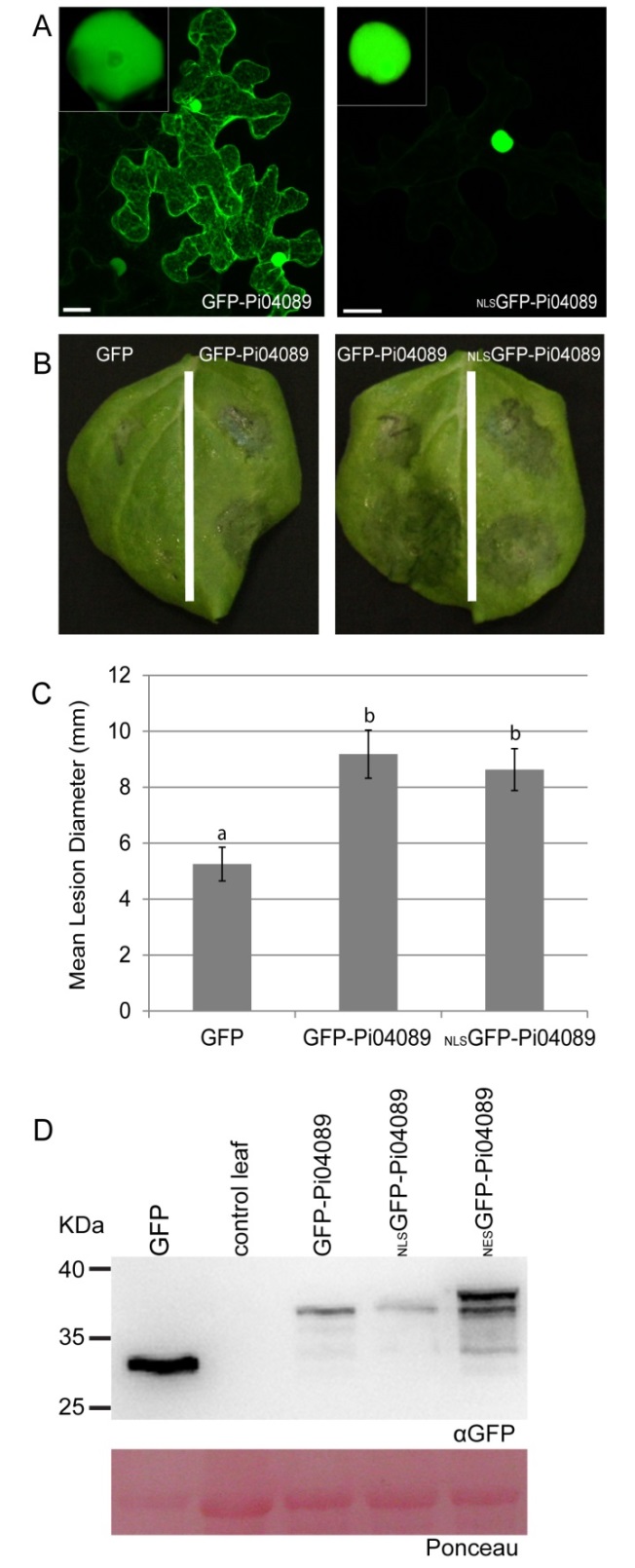
**

**Figure S2. The effect of an NLS on GFP-Pi04089.** (A) Images showing the increased nuclear fluorescence from _NLS_GFP-Pi04089 compared to GFP-Pi04089. Scale bar is 20 µm. (B) Leaves transiently expressing GFP-Pi04089, _NLS_GFP-Pi04089 and a GFP control infected with *P. infestans* 6 dpi after infection showing the increased lesion sizes with the effector fusions as compared to the GFP. (C) Graph of the mean diameters of lesions indicates that there was no significant difference (p= 0.6, ANOVA) between the increase in *P. infestans* growth caused by either GFP-Pi04089 or _NLS_GFP-Pi04089 compared to GFP (p<0.001, ANOVA). ). Error bars are standard error and the graph represents the combined data from 2 biological reps (n =86 per construct). (D) Immunoblot indicates the unmodified and modified fusion proteins are largely stable.


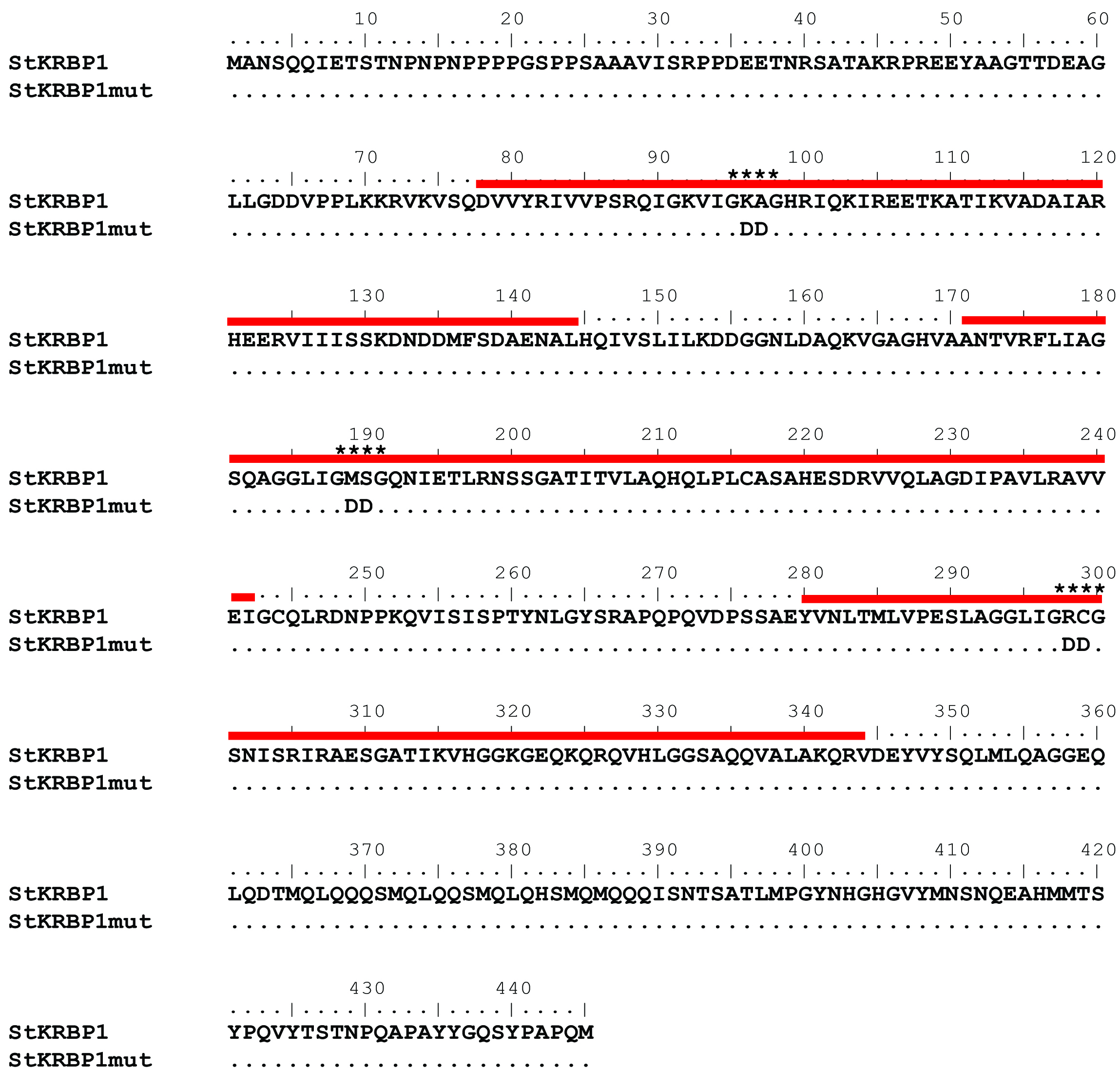


**Figure S3. Alignment of StKRBP1 and StKRBP1mut protein sequences.** Sequence of the StKRBP1, with the conserved KH domains indicated with red lines and the conserved GXXG motifs indicated with stars, aligned with the mutated form, showing only the changed residues.


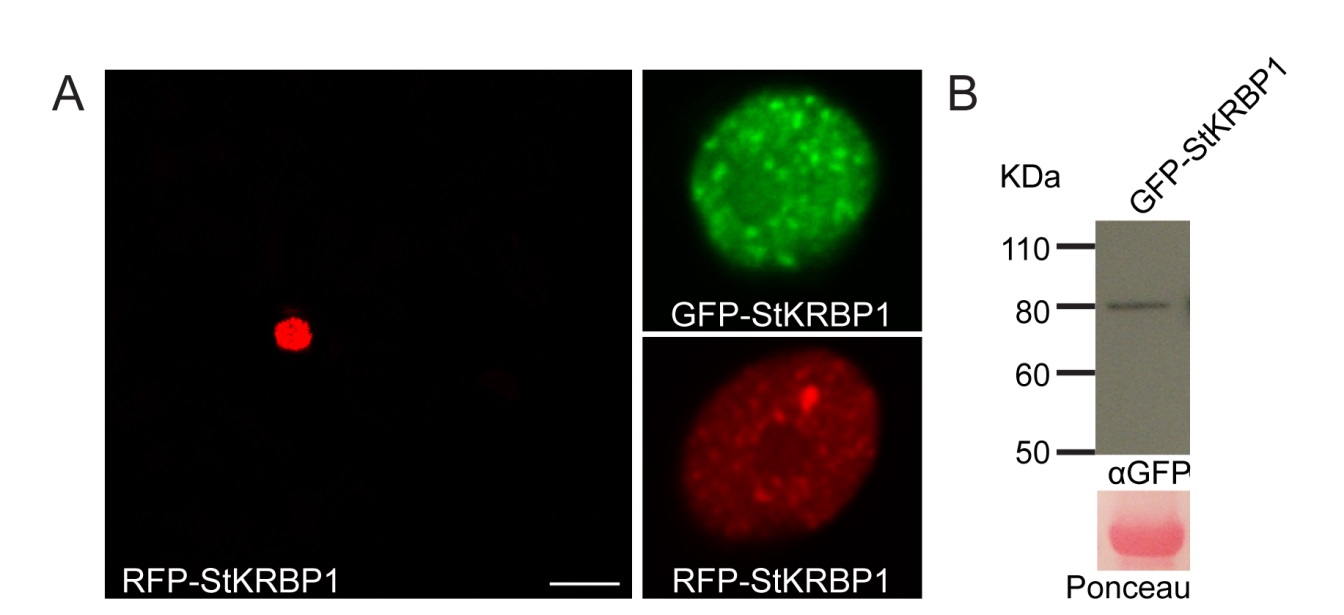


**Figure S4. Localisation and stability of StKRBP1 fusions.** (A) A low magnification image of RFP-StKRBP1 indicates fluorescence is only detected in the nucleus (left panel; scale bar represents 20μm). Single optical section images through nuclei expressing RFP-StKRBP1 and GFP-StKRBP1 showing localisation to nuclear speckles (right panels as indicated). (B) Immunoblot indicates the GFP-StKRBP1 protein fusion is stable.


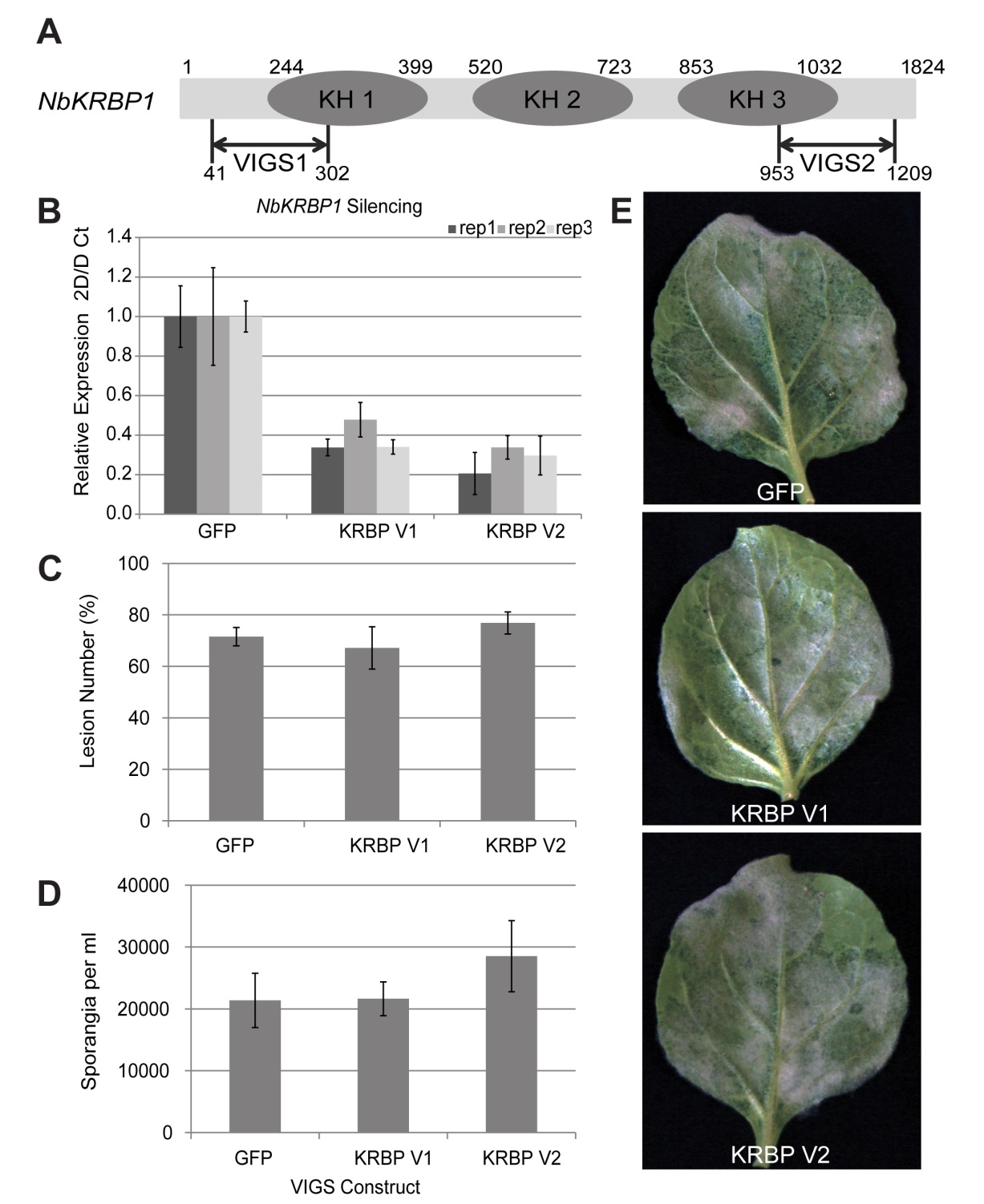


**Figure S5. VIGS of *NbKRBP1* has no effect on *P. infestans* virulence.** (A) A representation of the *NbKRBP1* with the nucleotide positions of three KH domains indicated by the numbers flanking the ovals. The locations of the sequences used for VIGS, VIGS1 and VIGS2 are shown. (B) RT PCR results on leaves from silenced plants from three biological replicates indicate significant reductions in the levels of *NbKRBP1* transcript with both VIGS constructs compared to control plants infected with TRV.GFP. (C) Graph showing that the number of lesions formed on silenced lines was not significantly different to control plants (p= 0.498, ANOVA). Error bars are standard error and the graph represents the combined data from 10 biological reps (n =10 per construct). (D) Graph showing that the numbers of sporangia per ml washed off infected leaves was also not significantly different for the silenced lines compared to the control (p= 0.442, ANOVA). Error bars are standard error and the graph represents the combined data from 6 biological reps (n =48 per construct). (E) Example leaf infections at 7 dpi.


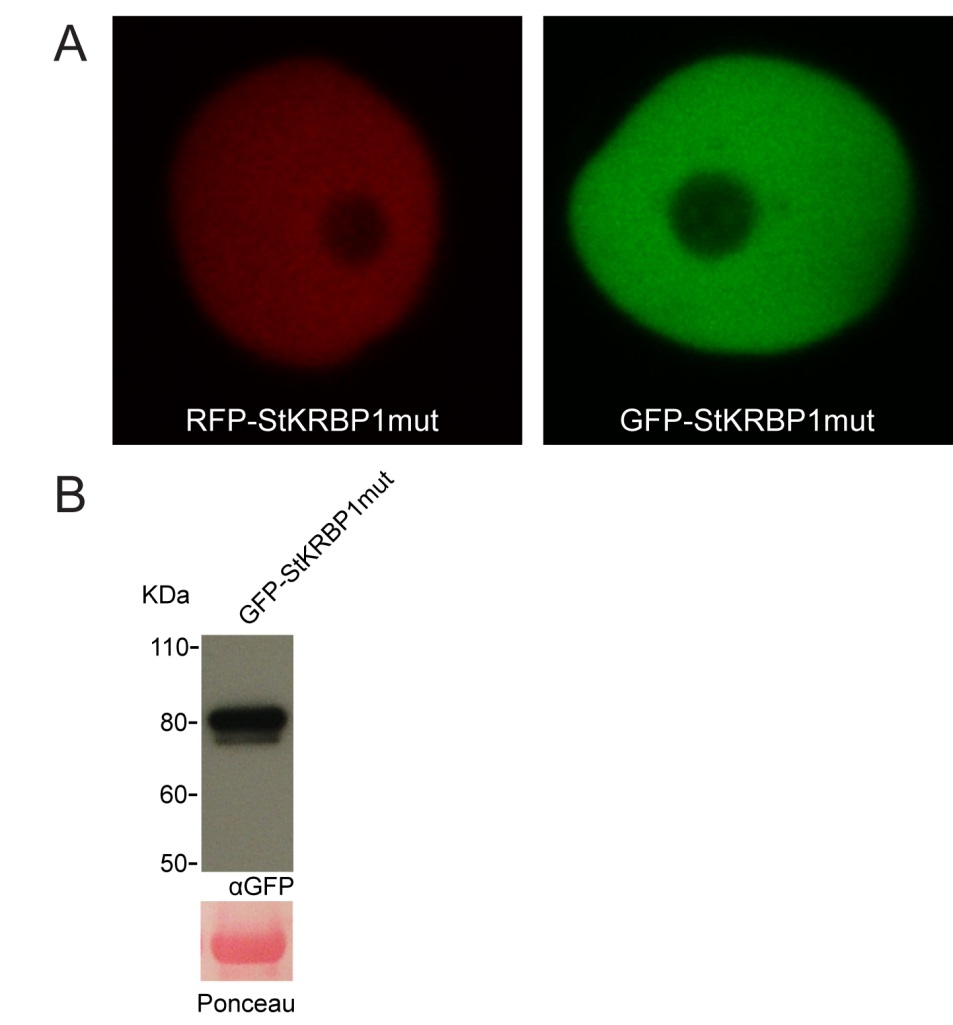


**Figure S6. Localisation and stability of StKRBP1mut fusions.** (A) Single optical section images through nuclei expressing tagRFP-StKRBP1mut or GFP-StKRBP1mut showing that the mutated forms do not locate to nuclear speckles but exhibit a smooth nucleoplasm localisation. (B) Immunoblot of the GFP fusion protein indicating that it is stable.

**Table S1.** **Table of all primers used in this study.**
